# Implant-Supported Overdenture Using Ball Attachments in Maxilla and Mandible: A Case Report

**DOI:** 10.7759/cureus.23264

**Published:** 2022-03-17

**Authors:** Sai Krishna, Rohan Kumar, Kunchala Sailasri

**Affiliations:** 1 Prosthodontics, SVS Institute of Dental Sciences, Mahabubnagar, IND; 2 Prosthodontics, SK Dental Hospital, Hyderabad, IND; 3 Prosthodontics and Crown and Bridge, SVS Institute of Dental Sciences, Mahabubnagar, IND

**Keywords:** implant, sleeves, ball and socket attachments, window preperation, alginate index, pickup impression, loading, attachments, overdenture, supported

## Abstract

Implant-supported overdentures are advantageous over conventional dentures, as they improve patient esthetics and enable retention, stability, comfort, and psychological well-being of the patient. This article describes a simple chairside technique for loading maxillary and mandibular dentures onto implant ball attachments.

## Introduction

Placing implants in completely edentulous patients may reduce the amount of bone resorption and enable the retention and stability of the prosthesis. It is difficult to place multiple implants in all edentulous areas because of compromised bone condition, but placing two or three implants in the maxilla within the premaxillary area and two implants in the mandible within the mental foramen is easy due to fewer anatomic limitations.

It is widely accepted that implant-supported overdenture is not the gold standard of implant therapy; rather, it is the minimum standard [1], which should be sufficient for most people, considering account performance, patient satisfaction, cost, and clinical time.

After six months of implant placement, ball attachments are incorporated over implants, which are parallel to each other, and chairside loading of maxillary and mandibular denture was performed using auto polymerizing acrylic resin.

## Case presentation

Patient information

A patient aged 48 who is well built came to the prosthodontics specialty clinic with a chief complaint of multiple missing teeth in both upper and lower jaws and some teeth are mobile due to gum problems. A treatment plan was clearly explained and patient consent was taken.

Clinical findings

Upon clinical examination, the patient had multiple missing teeth and some teeth are mobile due to poor periodontal status.

Diagnostic assessment

Based on clinical findings and relevant medical history, the patient was diagnosed with a partially edentulous maxilla and mandible with generalized periodontitis.

Therapeutic intervention

Upon clinical findings and diagnostic assessment, immediate implant placement was carried out followed by a prosthetic phase after seven months. The patient was recalled after the treatment and the outcome was found to be satisfactory.

Treatment

**Figure 1:**
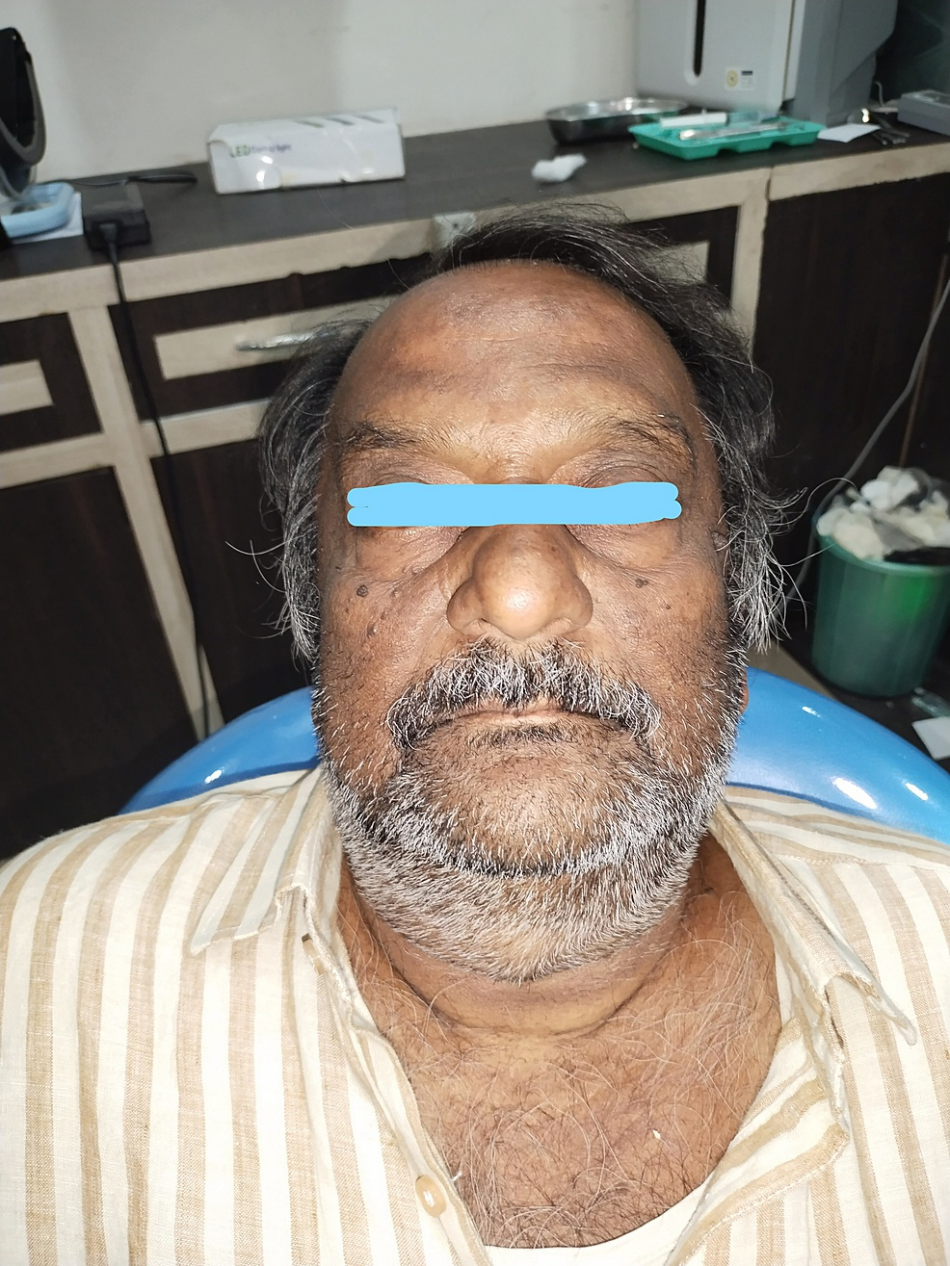
Preoperative image

A patient arrived at the clinic complaining of mobile and missing teeth. During the examination, it was discovered that the patient had multiple missing teeth and some teeth were mobile due to generalized periodontitis. A complete case history was obtained, and all extractions and immediate implant placement were planned (Figure 1).

Surgical Phase

Extractions were performed using extraction forceps with minimally invasive techniques, and all teeth mobile in the oral cavity were extracted, sockets were curetted, followed by implant placement.

Two implants were placed in the maxilla within the premaxillary region [2], and two implants were placed in the anterior part of the mandible between two mental foramina [3]. The flap was approximated using simple interrupted sutures, and the patient was recalled after two weeks for conventional denture fabrication and after six months for denture loading.

Prosthetic Phase

**Figure 2:**
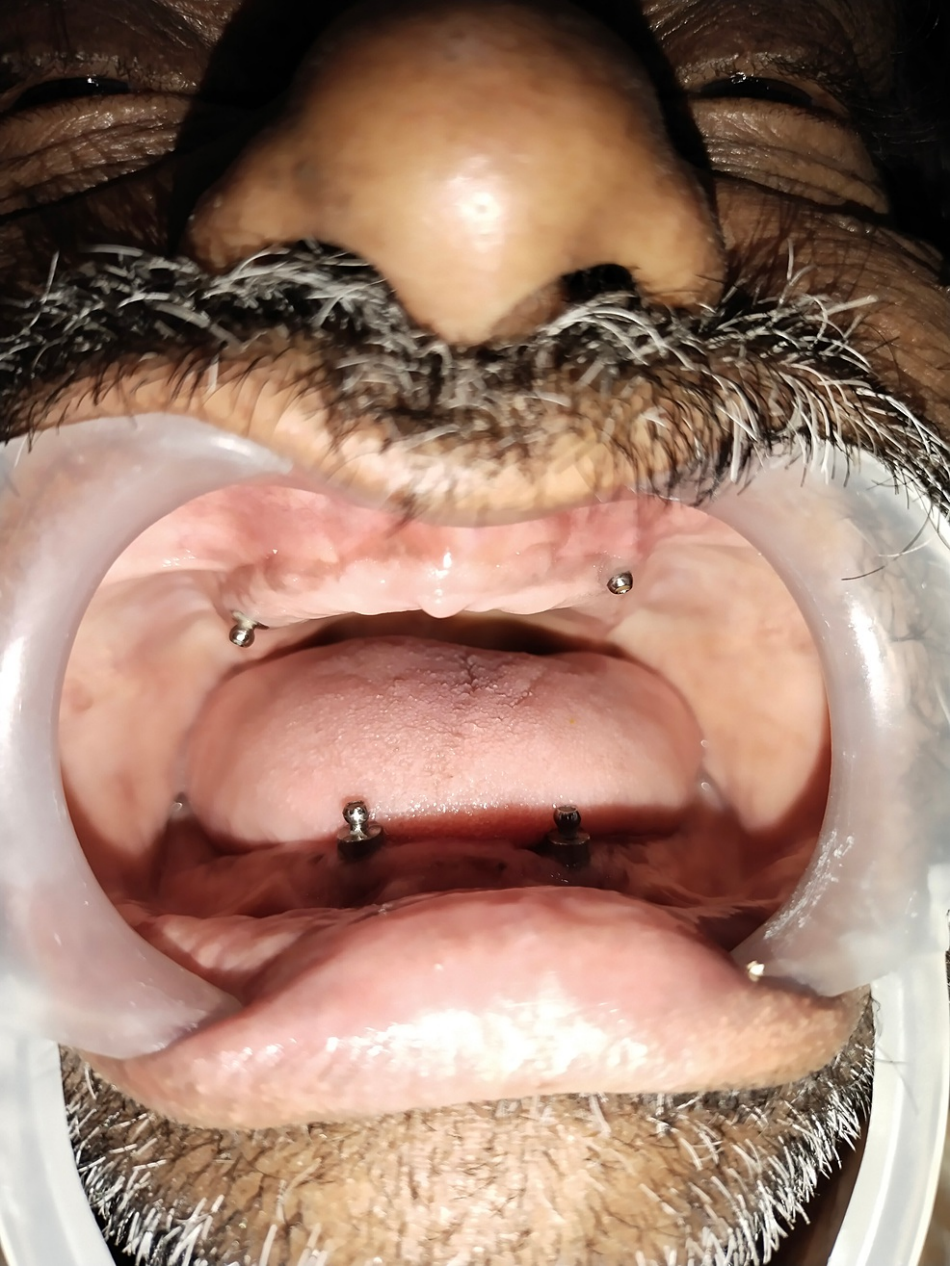
Implants with ball attachments

The patient was recalled after six months, and localized incisions were made to reflect the flap, implants were located, and healing abutments were fixed on all implants for two weeks [4]. After two weeks, the patient was recalled, and healing abutments were replaced with the ball [5] attachments (Figure 2).

**Figure 3:**
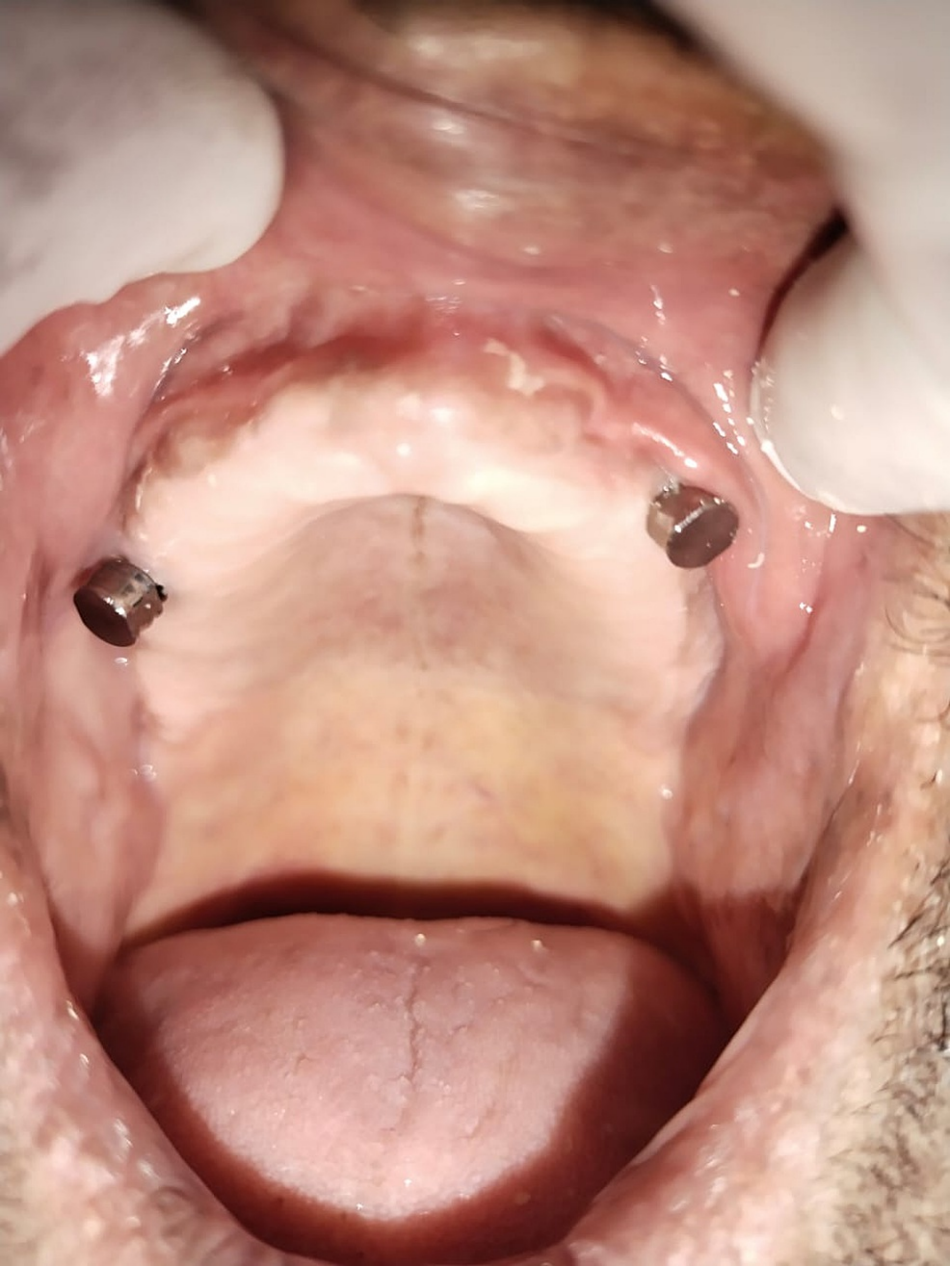
Metal housings over ball attachments

**Figure 4:**
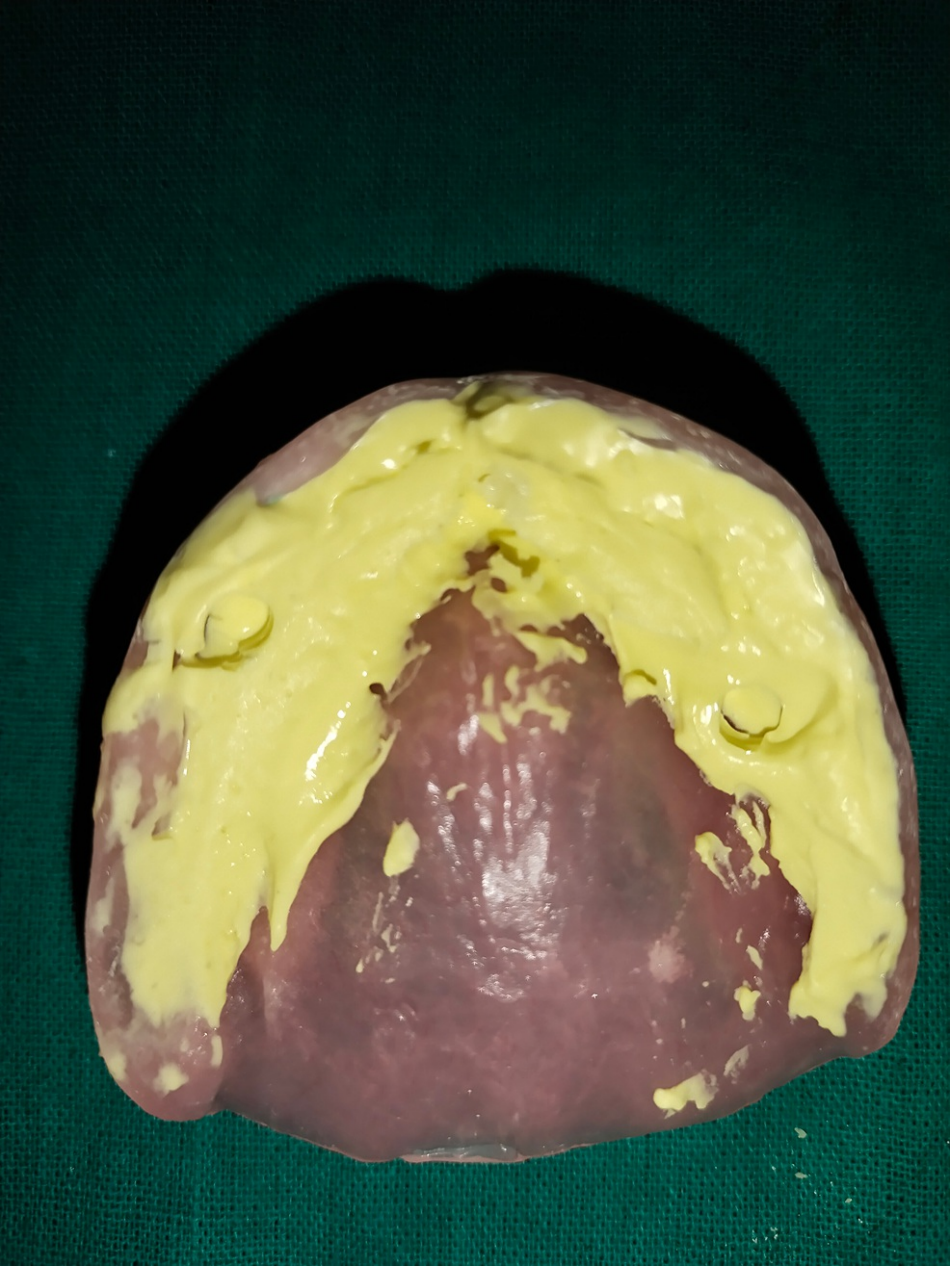
Alginate index for housings locations

An index was made using alginate for ball attachment locations on the intaglio surface of a denture, and a hallow was made in that area to receive female housings (Figures 3, 4).

**Figure 5:**
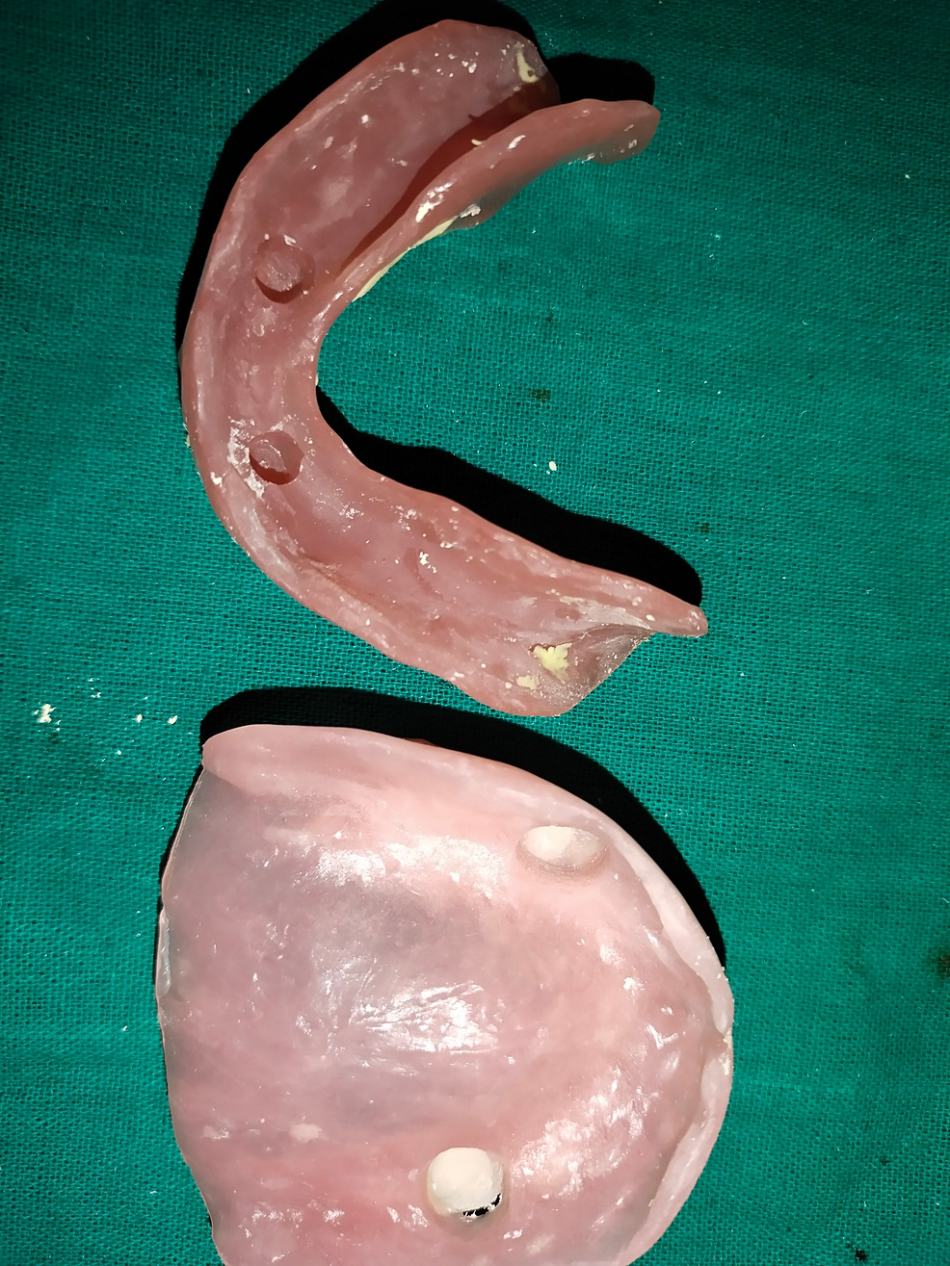
Hallow preparation on tissue surface of dentures

A rubber dam was cut and placed around the ball attachment on the tissue to prevent tissue injury during acrylic polymerization. Female housings were incorporated over male ball attachments [6], which are held parallel to each other in the parallel path of the axis. The self-cure acrylic resin was mixed and injected into the hollow space created on the tissue surface for both maxillary and mandibular dentures, and both dentures were positioned inside the patient’s mouth, and the patient was asked to bite in centric occlusion (Figure 5).

**Figure 6:**
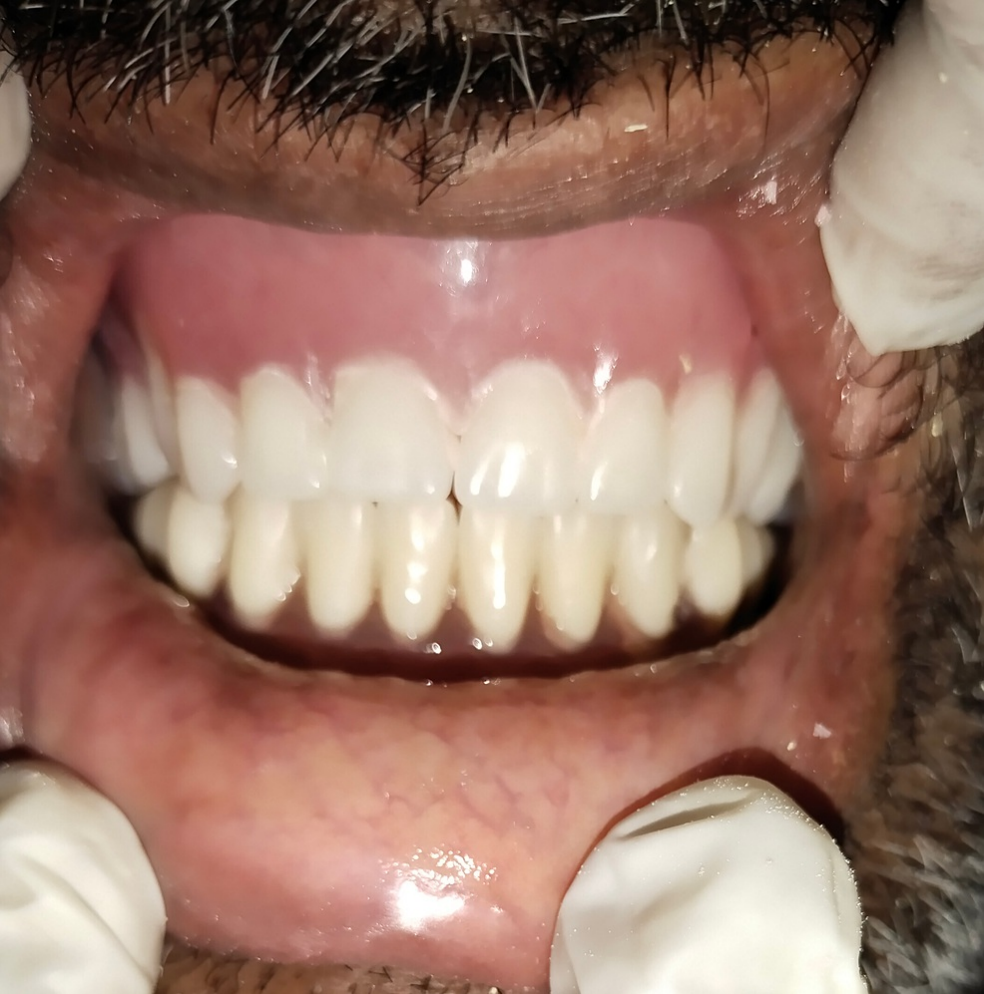
Denture insertion over ball attachments

The material was allowed to be set for some time and was removed from the mouth. Excess materials were trimmed and finished before being reoriented in the same position intraorally (Figure 6).

## Discussion

Implant-supported overdenture is a predictable treatment option that provides patients with better retention and stability for a prosthesis. Placing two or three implants in the maxilla or mandible will yield the best clinical [6] outcome.

Implants should be prescribed based on clinical diagnoses and the need of patients, rather than the preference of the clinician. The implant-supported overdenture is a simple treatment option for both the patient and doctor because it is economical (affordable to most patients) and requires fewer visits to complete the treatment.

In this case report, the patient arrived at the clinic with a completely edentulous maxilla and mandible. On clinical and radiographic examination, the patient has flabby mucosa and clinically well-contoured ridges. Radiographically, the patient has good bone support in the maxilla anterior region and mandibular anterior and posterior regions through CBCT interpretation. The patient has less bone in the maxilla posterior region because the sinus is close to the crest of the ridge. To avoid all extensive surgical procedures, the patient was advised to have two implants placed in both the maxilla and mandible, followed by an implant-supported [7] overdenture.

Two implants were placed in both the maxilla and mandible. Thereafter, the patient was recalled after seven months for the second stage. During the second stage, implants were checked for stability and healing abutments were fixed in their positions. The patient was recalled after one week and ball attachments were incorporated in place of healing abutments, and the index was made using alginate.

The alginate index acts as a guide for the preparation of hollow spaces on the tissue surface of a previous denture in order to receive metal housings. Upon preparation, metal housings are picked using the chairside pickup technique with a self-cure resin [8]. After chairside pickup and occlusion were evaluated and corrected for occlusal interferences, proper trimming and finishing were done.

## Conclusions

Although an implant-supported overdenture is a minimum standard treatment option, various clinicians have used it to overcome clinically compromised situations and the cost of treatment. Implant-supported overdenture provides the patient with good retention and stability for a prosthesis, as well as psychological well-being as a fixed prosthesis.
